# Benchmarking Zero-Shot Open-Vocabulary and Fine-Tuned Object Detectors for Underground Mine Personnel Detection

**DOI:** 10.3390/s26175646

**Published:** 2026-09-05

**Authors:** Ellen Essien, Samuel Frimpong

**Affiliations:** Department of Mining and Explosives Engineering, Missouri University of Science and Technology, Rolla, MO 65409, USA; frimpong@mst.edu

**Keywords:** underground mining, personnel detection, object detection, vision-language models, YOLO, autonomous haulage, mine safety and health

## Abstract

**Highlights:**

**What are the main findings?**

- Fine-tuned YOLO detectors substantially outperformed zero-shot vision-language models for underground mine personnel detection.
- YOLO26s achieved the strongest overall supervised performance, while Grounding DINO was the strongest zero-shot detector.

**What are the implications of the main findings?**

- Domain-specific fine-tuning remains important for reliable underground personnel detection under challenging mining conditions.
- The benchmark quantifies accuracy, localization, computational performance, condition-specific, and target-scale differences between supervised and zero-shot detection paradigms.

**Abstract:**

Reliable personnel detection is critical for the safe deployment of autonomous haulage systems in underground mining, where challenging environmental conditions demand robust real-time perception. Existing research has focused primarily on fine-tuned convolutional detectors, while systematic comparisons with zero-shot vision-language models remain limited. This study presents a cross-paradigm benchmark comparing four zero-shot vision-language models (YOLO-World, Grounding DINO, OWL-ViT, and OWLv2) with four fine-tuned YOLO detectors (YOLOv8s, YOLOv9s, YOLO11s, and YOLO26s) using 31,396 real-world underground coal mine images. Detection performance was evaluated using precision, recall, F1-score, average precision, inference speed, and condition- and target scale-specific recall. The experimental results show that the fine-tuned detectors achieved F1-scores of 0.8449–0.8662 and AP50 values of 0.8791–0.9135, with YOLO26s achieving the strongest overall performance. In comparison, the zero-shot models achieved F1-scores of 0.3186–0.5484 and AP50 values of 0.2484–0.5108, with Grounding DINO performing best among the zero-shot models. Fine-tuned detectors also maintained substantially higher recall for occluded personnel and small apparent targets. These findings demonstrate a substantial performance advantage for domain-specific fine-tuning over the evaluated zero-shot approaches and establish a controlled cross-paradigm benchmark for comparing detection paradigms for underground personnel perception.

## 1. Introduction

Autonomous haulage systems (AHS) represent one of the most significant technological advances in contemporary mining, improving operational productivity and worker safety. Commercial autonomous haulage fleets have demonstrated significant operational advantages in surface mining, driving increasing interest in extending autonomous operations to underground mining environments [1,2]. However, underground mining environments introduce additional safety and perception challenges for autonomous haulage systems [3]. Reliable perception is therefore critical for real-time personnel detection, preventing vehicle–worker collisions, and supporting safe autonomous operation. Underground environments present adverse conditions that can markedly impair visual perception, including low and non-uniform illumination, airborne dust, motion blur, equipment occlusion, water mist, and confined tunnel geometries, which may occur simultaneously [4]. Such conditions reduce object visibility and introduce substantial domain shifts between underground imagery and the natural-image datasets commonly used to train modern computer vision models [5]. This degradation can increase missed detections and false alarms, reducing the reliability of safety-critical personnel perception. Developing robust personnel detection models that maintain reliable performance under these conditions is therefore important for advancing underground autonomous haulage systems.

Recent advances in deep learning have significantly enhanced object detection across several industrial domains, including intelligent transportation, manufacturing, construction safety, and mining [6,7,8,9]. In underground mine personnel detection, many studies rely on convolutional neural network (CNN) detectors, especially successive versions of the You Only Look Once (YOLO) architecture, due to their favorable trade-off between detection accuracy and real-time inference speed [10,11,12,13,14]. More recently, large-scale vision-language models (VLMs) have introduced an alternative paradigm for object detection. Models such as Grounding DINO, OWL-ViT, OWLv2, and YOLO-World use visual-semantic representations learned from large-scale image–text datasets to enable open-vocabulary object detection without domain-specific retraining [15,16,17].

Despite rapid progress in fine-tuned CNN detectors and zero-shot vision-language models, their relative capabilities for underground mine personnel detection remain largely unexplored. Existing underground mining studies have primarily evaluated proposed CNN architectures against other closed-set detectors, while modern zero-shot vision-language models have received limited evaluation in this domain. Differences in datasets, evaluation protocols, confidence thresholds, and reported performance metrics across previous studies further limit direct comparison between detection approaches. As a result, there is limited evidence on how fine-tuned and zero-shot detectors compare in detection performance, localization quality, and computational performance under underground mining conditions. This gap motivates a systematic cross-paradigm evaluation of both detection approaches under a common experimental framework.

To address this gap, this study presents a systematic cross-paradigm benchmark for underground mine personnel detection. The benchmark compares four zero-shot vision-language models (YOLO-World, Grounding DINO, OWL-ViT, and OWLv2) with four fine-tuned YOLO detectors (YOLOv8s, YOLOv9s, YOLO11s, and YOLO26s) using a common dataset of 31,396 real-world underground coal mine images. To reduce information leakage from closely related neighboring images, the benchmark uses a sequence- and provenance-aware grouped train–validation–test partition. Both paradigms are evaluated under a unified experimental framework using the same held-out test set, common detection and localization metrics, and a hardware-controlled computational environment. For the zero-shot models, model-specific operating confidence thresholds are selected exclusively on the validation subset and frozen before final test evaluation. These experimental controls provide a consistent and reproducible basis for comparing detection performance across the two paradigms, while the common GPU configuration provides a hardware-controlled reference for computational performance. The resulting benchmark provides quantitative evidence of the relative capabilities and limitations of fine-tuned and zero-shot detectors for underground personnel detection.

The primary objective of this study is to quantify performance differences between fine-tuned YOLO detectors and zero-shot vision-language models for detecting underground mine personnel within a common experimental framework. Specifically, the study evaluates the two detection paradigms using precision, recall, F1-score, average precision, inference speed, and condition- and target scale-specific recall. This evaluation assesses how effectively current zero-shot detection capabilities transfer to the visually challenging underground mining domain relative to detectors fine-tuned on domain-specific imagery, and it identifies the operating conditions and target scales where performance differences between the two paradigms are most pronounced.

The major contributions of this study are summarized as follows:

- We present a controlled cross-paradigm benchmark comparing fine-tuned YOLO detectors and zero-shot vision-language models for detecting underground mine personnel under a common experimental framework.
- We evaluate eight state-of-the-art detection models using a common dataset from an underground coal mine, standardized performance metrics, and a unified evaluation protocol to ensure fair and reproducible comparisons across learning paradigms.
- We characterize cross-paradigm differences in detection accuracy, localization performance, computational performance under common benchmark hardware, condition-specific recall, and target-scale sensitivity, providing quantitative evidence of the relative strengths and limitations of supervised and zero-shot detection under underground mining conditions.
- We establish benchmark baselines that support future research and the development of reliable AI-based perception systems for autonomous underground mining.

The remainder of this paper is organized as follows. Section 2 reviews recent advances in underground mine personnel detection, zero-shot vision-language models, and cross-paradigm benchmarking studies. Section 3 describes the underground coal mine dataset, benchmark models, experimental setup, evaluation protocol, and performance metrics. Section 4 presents the benchmarking results and compares the performance of zero-shot vision-language models with fine-tuned YOLO detectors. Section 5 discusses the implications of the findings for underground autonomous haulage systems, highlights the strengths and limitations of the evaluated detection paradigms, and outlines future research directions. Section 6 concludes by summarizing the study’s major findings and contributions.

## 2. Existing Knowledge

This section reviews the literature underpinning the present study, with emphasis on three areas: YOLO-based approaches for underground mine personnel detection, zero-shot vision-language models for object detection, and benchmarking studies comparing supervised and zero-shot detection approaches. The review examines the development, performance, and limitations of these approaches and identifies the methodological and application-specific gaps that motivate the cross-paradigm benchmark developed in this study.

### 2.1. Deep Learning-Based Detection Models

Coal mine safety has long been a concern for mining operations worldwide, and personnel-vehicle proximity incidents are among the most critical hazards in underground mining [18,19]. The increasing use of autonomous and semi-autonomous mobile equipment has created a greater need for reliable personnel detection. Such systems must detect mine personnel in real time to support collision avoidance and safety-critical decision making. This need has driven research on deep learning-based object detection for underground mining environments.

Over the past decade, the YOLO family has emerged as a widely adopted framework for real-time object detection because of its favorable balance between detection accuracy and computational efficiency [20]. Unlike two-stage detectors, which first generate region proposals and then classify candidate objects, YOLO performs object localization and classification within a single-stage detection framework [20,21]. Successive YOLO generations have introduced changes in network architecture, feature extraction, training strategies, and prediction mechanisms. YOLOv8 adopted an anchor-free detection head and decoupled prediction architecture [22]. YOLOv9 introduced Programmable Gradient Information (PGI) to improve gradient flow and information preservation during training [23]. YOLO11 incorporated the C3k2 module and Cross-Stage Partial with Partial Self-Attention (C2PSA) to strengthen feature extraction and representation [24]. More recently, YOLO26 introduced native end-to-end detection without non-maximum suppression (NMS), together with Progressive Loss Balancing and the MuSGD optimization strategy [25,26]. These developments reflect the continued evolution of YOLO toward more efficient feature representation, training, and inference.

Early underground mining studies adapted CNN-based detectors to address the visual and computational challenges of mine environments. Li et al. [27] developed an improved YOLOv4 model for underground personnel detection using thermal infrared imagery. The method incorporated super-resolution preprocessing and K-means++ anchor-box optimization. The model achieved an average precision of 96.25% at 48.2 FPS, demonstrating the potential of single-stage detection for real-time underground safety monitoring. Zhou et al. [28] proposed UCM-Net, a YOLO-based detector with a custom ESFENet backbone and self-supervised pre-training. The model was designed to address computational constraints and complex underground operating conditions. Yang et al. [5] combined a Zero-DCE image enhancement module with YOLOv5s to improve personnel detection under poor illumination.

More recent studies have focused on improving YOLO-based detection under challenging underground visual conditions. Liu et al. [29] proposed YOLOv8-HAC for safety helmet detection under strong illumination, low-light conditions, and equipment occlusion in underground coal mines. Jin et al. [30] developed KD-YOLO, an improved YOLOv8-based personnel detector designed to address unclear personnel contours, occlusion, and small target sizes. The model reduced the number of parameters by 13.3% while improving detection performance. Similarly, Zhang et al. [31] proposed CGSW-YOLO, a YOLOv8n-based model for simultaneous personnel and vehicle detection in mine air-door environments. The model incorporated partial convolution to improve computational efficiency while maintaining real-time detection capability.

Recent research has also extended underground safety detection to YOLO11-based architectures. Zhang et al. [32] introduced GCB-YOLOv11, an enhanced version of the YOLOv11n architecture that integrates GSConv, a C3K2_FE module combining Faster-block and ECA attention mechanisms, along with a BiFPN neck. This model achieves a mean average precision of 93.6% at a processing speed of 90.3 FPS, enabling the concurrent detection of mine personnel and safety helmets under uneven lighting and equipment obstructions in fully mechanized mining environments. Li et al. [33] proposed an improved YOLOv11 model for underground mine personnel detection. The model was evaluated against SSD, YOLOX, Faster R-CNN, and standard YOLOv8 using a common underground mine dataset. Their results showed that the proposed model outperformed the evaluated baseline detectors.

The existing literature demonstrates substantial progress in deep learning-based underground personnel detection. However, most studies rely on domain-specific supervised training and primarily evaluate proposed models against other closed-set detectors. Comparisons of recent YOLO generations within a consistent experimental framework remain limited, making it difficult to assess their relative performance. More importantly, modern zero-shot vision-language models have received limited evaluation for underground personnel detection, particularly in direct comparison with fine-tuned detectors. This leaves an important gap in understanding how supervised and zero-shot detection approaches compare under challenging underground conditions. The present study addresses this gap through a systematic cross-paradigm benchmark of fine-tuned YOLO detectors and zero-shot vision-language models for underground mine personnel detection.

### 2.2. Zero-Shot Vision-Language Models for Object Detection

The emergence of large-scale vision-language pre-training has introduced a new approach to object detection based on visual-semantic learning. Unlike conventional detectors that require task-specific training, vision-language models (VLMs) learn joint visual-semantic representations from large-scale image-text datasets. These representations enable models to detect objects specified through text prompts without task-specific fine-tuning [34]. This capability is particularly attractive in industrial applications where acquiring and annotating diverse training datasets can be expensive, time-consuming, and operationally difficult.

Radford et al. [34] introduced CLIP, which laid the foundation for zero-shot visual recognition by learning aligned image and text representations through contrastive training on 400 million image–text pairs. Liu et al. [15] extended this vision-language paradigm to object detection with Grounding DINO. The model combines transformer-based detection with language grounding to localize objects specified by text prompts. Minderer et al. [16] introduced OWL-ViT, which adapts a Vision Transformer for open-vocabulary object detection using text queries. OWLv2 later extended this framework through large-scale self-training to improve open-vocabulary detection performance [35]. Cheng et al. [17] proposed YOLO-World for real-time open-vocabulary detection. The model integrates vision-language representations into a YOLO-based architecture, combining semantic flexibility with the computational efficiency of single-stage detection.

These architectures have since been applied to several industrial safety tasks. Choi and Greer [36] used OWLv2 in a cascaded detection pipeline for zero-shot hardhat detection and worker–hardhat association on construction sites. Wu et al. [37] developed MonitorVLM to identify safety-rule violations from observed worker behaviors. The framework was evaluated using surveillance footage from surface and underground mining operations. Abdullah et al. [38] introduced iSafetyBench, a benchmark comprising industrial video clips and hazardous-action categories. The benchmark evaluates zero-shot hazard recognition across multiple vision-language models and industrial settings.

Although zero-shot vision-language models have demonstrated promising performance across natural-image and industrial applications, their performance for underground mine personnel detection remains largely unexplored. Existing robustness studies indicate that open-vocabulary detectors such as OWL-ViT, YOLO-World, and Grounding DINO can be sensitive to image corruption and distribution shifts [39]. This is relevant to underground mining, where poor illumination, reduced visibility, and occlusion can degrade visual information. Yet, evidence on the performance of modern zero-shot detectors for underground personnel detection remains limited. Direct comparisons with domain-specific fine-tuned detectors are also limited. These gaps motivate the cross-paradigm evaluation conducted in this study.

### 2.3. Cross-Paradigm Benchmarking Studies

Systematic benchmarking has played a fundamental role in advancing object detection by providing standardized datasets, evaluation protocols, and performance metrics for objective model comparison. Widely adopted benchmarks such as Pascal VOC [40] and Microsoft COCO [41] have established common evaluation frameworks that have driven significant improvements in detection accuracy, localization performance, and computational efficiency. Beyond general computer vision, domain-specific benchmarks provide standardized frameworks for comparing model performance under application-specific environmental and operational conditions.

Recent studies have compared zero-shot and fine-tuned detection approaches across general computer vision, multi-domain benchmarks, and specialized applications. Robicheaux et al. [42] introduced Roboflow100-VL, a benchmark spanning 100 visual domains. The benchmark evaluates vision-language models under zero-shot, few-shot, semi-supervised, and fully supervised settings. Their results showed that zero-shot models generally performed below fine-tuned models. Performance also declined in out-of-distribution domains such as medical imaging. In contrast, Madan et al. [43] reported stronger zero-shot performance on the COCO benchmark. Grounding DINO in zero-shot mode outperformed several state-of-the-art few-shot detectors. These contrasting findings indicate that relative model performance can vary across domains and evaluation settings. Sapkota et al. [44] extended this comparison to an agricultural application. They evaluated the Segment Anything Model (SAM3) against three fine-tuned YOLO11 variants on a densely populated orchard dataset. Their results showed that the fine-tuned YOLO11 models achieved higher instance-level F1-scores than zero-shot SAM3 on a densely populated orchard dataset characterized by high object density and occlusion.

Current cross-paradigm benchmarking studies remain limited in underground mining applications. Existing work has focused mainly on natural-image datasets, broad multi-domain benchmarks, and specialized industrial or agricultural applications. Direct comparisons between zero-shot vision-language models and fine-tuned detectors for underground personnel detection remain scarce. This gap is important because poor illumination, airborne dust, motion blur, and equipment occlusion can reduce personnel visibility and increase detection difficulty. Limited evidence is available on how the two detection paradigms perform under these conditions. Their relative detection performance, localization capability, and computational performance also remain unclear for underground personnel detection. The present study addresses this gap through a standardized cross-paradigm benchmark of four zero-shot vision-language models and four fine-tuned YOLO detectors.

## 3. Benchmark Methodology

**Figure 1:**
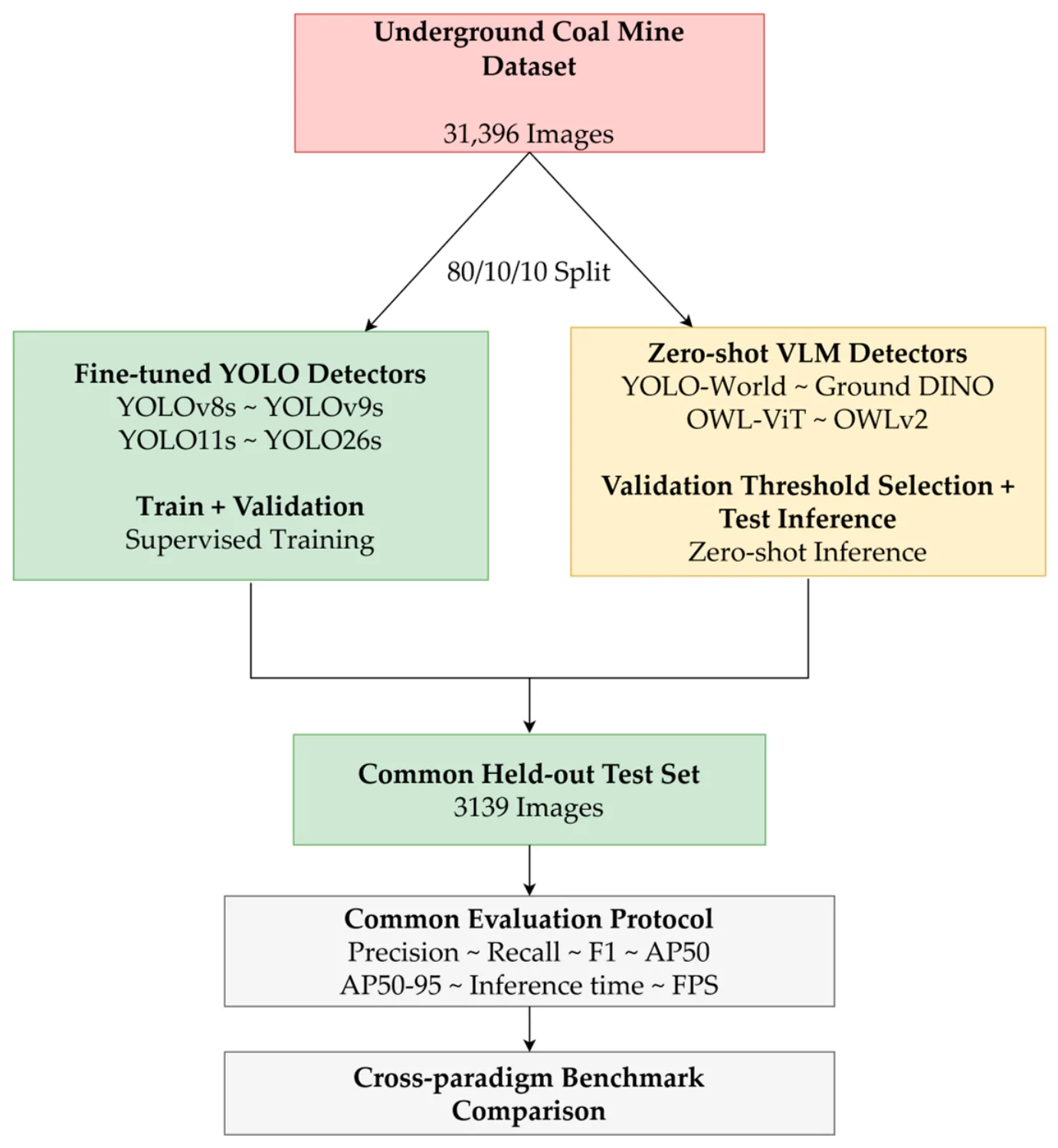
Overall benchmarking framework for comparing fine-tuned YOLO detectors and zero-shot vision-language models.

This section outlines the methodology for benchmarking fine-tuned YOLO detectors and zero-shot vision-language models for detecting underground mine personnel. The methodology covers dataset preparation, benchmark models, experimental configuration, and evaluation protocol. Figure 1 presents the overall benchmarking framework and the main stages of the experimental workflow.

### 3.1. Underground Coal Mine Dataset

A publicly accessible dataset of underground coal mine images was used for the benchmark evaluation [45]. The original dataset comprises 70,948 RGB images captured during operational drilling in underground coal mines at a resolution of 1280 × 720 pixels. The images are annotated in Pascal VOC XML format across five object categories: coal_miner, mine_safety_helmet, gripper, drill_pipe, and chuck. Because this study focuses on underground personnel detection, only annotations corresponding to the *coal_miner* class were retained. Images containing no annotated miner after class filtering were excluded from the benchmark. This process resulted in 31,396 miner-positive images. These images formed the single-class personnel detection dataset used in this study. The retained annotations were converted to YOLO format for supervised model training and benchmark evaluation.

**Figure 2:**
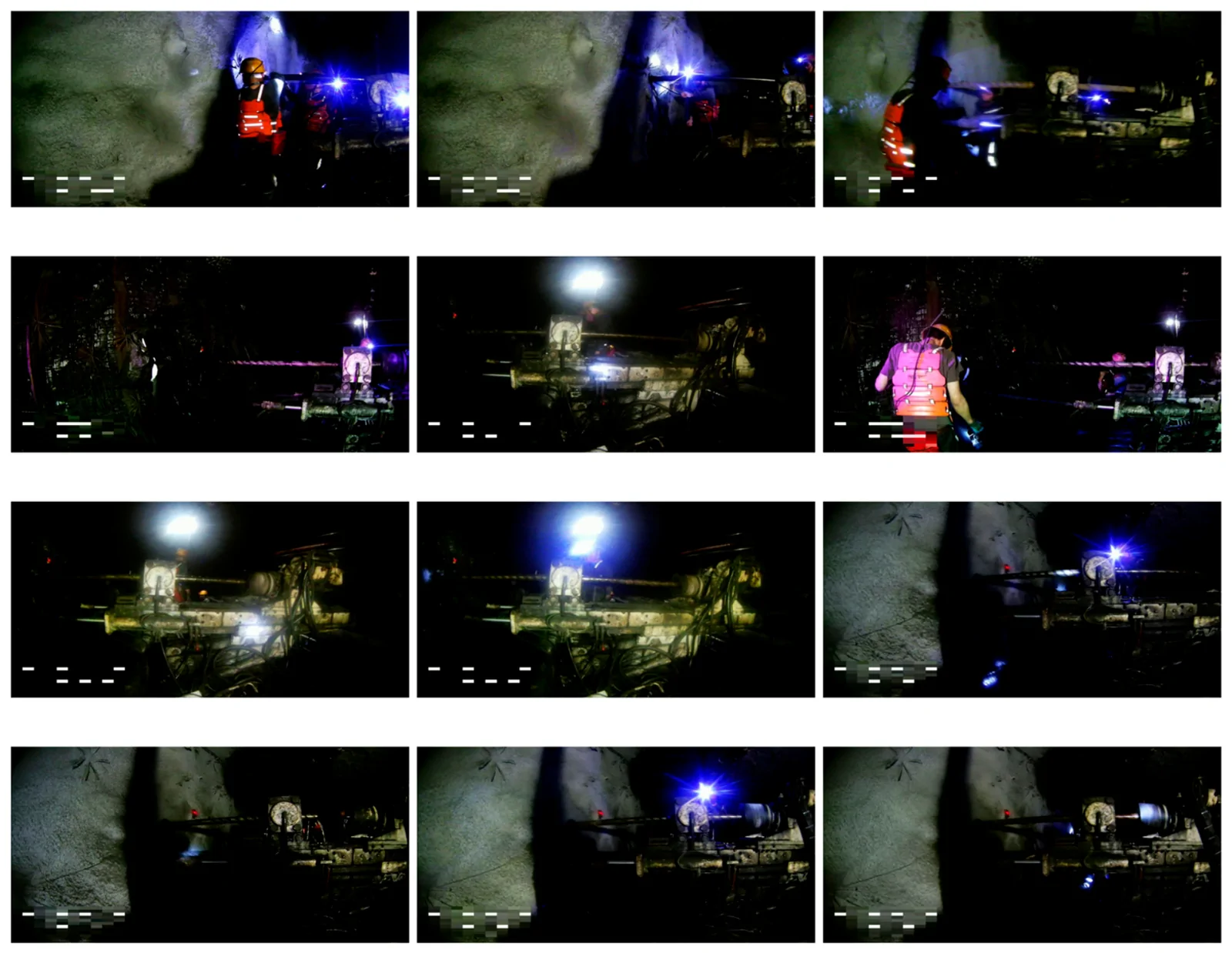
Representative images from the underground coal mine personnel detection dataset.

The dataset exhibits substantial variation in personnel appearance and underground scene characteristics. Miner pose, viewing angle, apparent target size, and level of occlusion vary across images. The scenes also show illumination and visibility changes associated with underground operations. These include low and non-uniform lighting, bright local light sources, image blur, equipment occlusion, and visually cluttered backgrounds. The dataset therefore captures substantial variation in underground visual conditions, providing a suitable basis for evaluating detector performance across different operating scenarios. Representative images are shown in Figure 2.

A grouped splitting strategy was used to reduce information leakage from closely related images. The split was reconstructed from the complete 70,948-image dataset before personnel-class filtering. Source groups were identified using contiguous image-index sequences and the provenance information available in the original annotation paths. Images associated with the same reconstructed source group were assigned to only one dataset subset. This procedure ensured that closely related images from the same source sequence were assigned to only one of the training, validation, or test subsets. Verification of the final partition identified 244 source groups containing miner instances, with no source group shared across subsets. The filtered dataset was then divided into training, validation, and test subsets using an approximately 80:10:10 allocation. The resulting partition contains 25,117 training images, 3140 validation images, and 3139 test images. The training subset was used to fit the supervised YOLO detectors. The validation subset was used for model selection and parameter tuning. For the zero-shot models, the validation subset was also used to select model-specific operating confidence thresholds before test evaluation. The test subset was reserved for the final comparison of all detection models. It contains 3139 images and 3408 annotated miner instances. Table 1 summarizes the final dataset partition.

### 3.2. Benchmark Models

**Figure 3:**
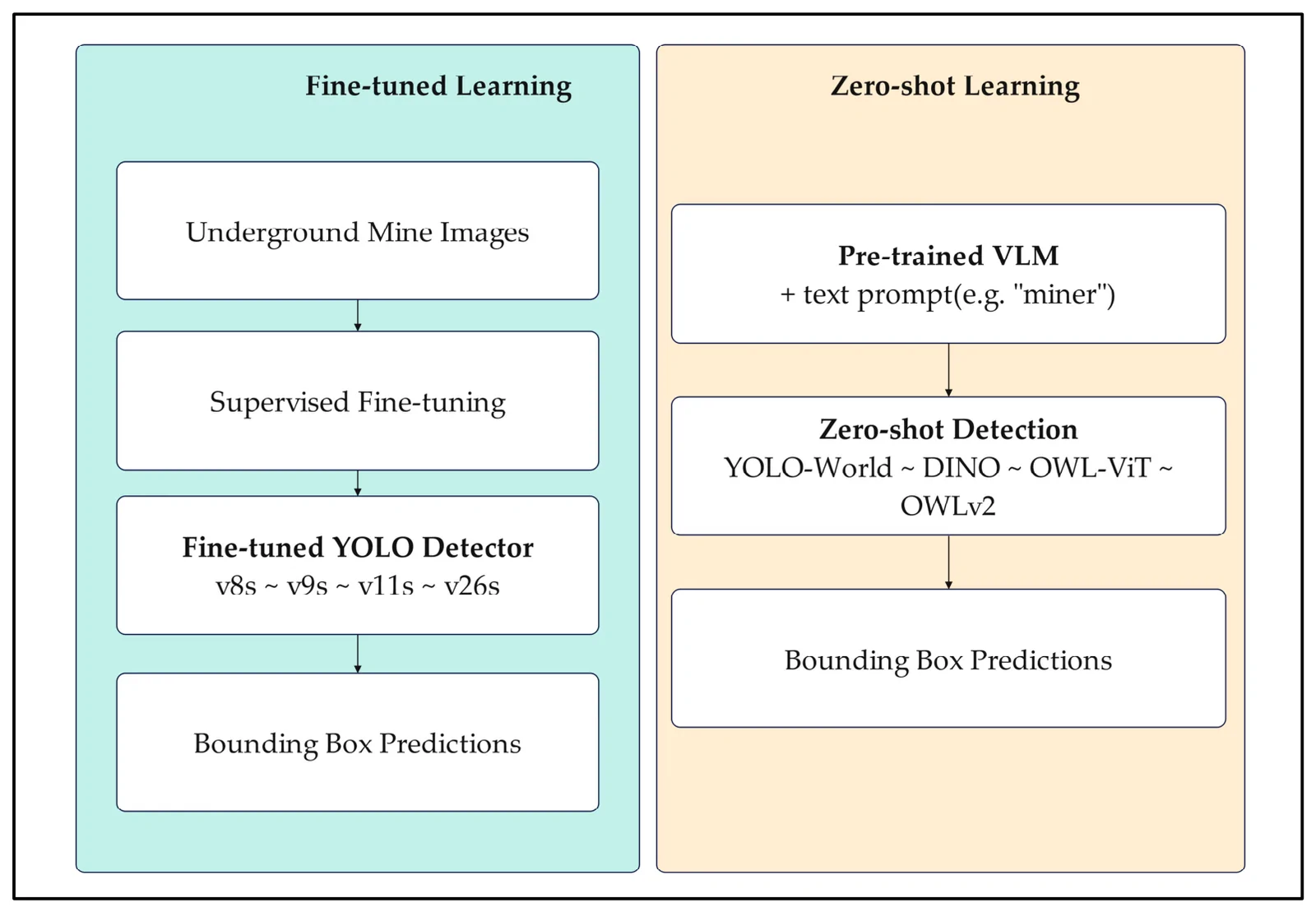
Comparison of fine-tuned and zero-shot detection paradigms for underground personnel detection.

This study compares two object detection paradigms: fine-tuned supervised detectors and zero-shot vision-language models. The fine-tuned detectors learn task-specific representations from labeled underground mine images. In contrast, the zero-shot models use pretrained visual-semantic representations and natural-language prompts to localize personnel without training on the benchmark dataset. Figure 3 illustrates the conceptual distinction between the two paradigms.

Eight models were selected for the benchmark. The supervised group comprises YOLOv8s, YOLOv9s, YOLO11s, and YOLO26s. The zero-shot group comprises YOLO-World, Grounding DINO, OWL-ViT, and OWLv2. The selected models represent recent architectures with different feature-extraction, prediction, and visual-semantic modeling strategies. Table 2 summarizes the models and their principal architectural characteristics. Table 3 and Table 4 provide more detailed comparisons within the supervised and zero-shot groups, respectively.

The supervised benchmark includes four small-scale detectors from successive YOLO generations: YOLOv8s, YOLOv9s, YOLO11s, and YOLO26s. The small variants were selected to provide broadly comparable lightweight model configurations while capturing architectural progression across successive YOLO generations. YOLOv8 introduced an anchor-free detection framework with C2f-based feature extraction [22]. YOLOv9 incorporated the Generalized Efficient Layer Aggregation Network (GELAN) and Programmable Gradient Information (PGI) [23]. YOLO11 introduced C3k2 and C2PSA modules for feature representation [24]. YOLO26 further introduced native end-to-end NMS-free inference and DFL-free box regression. Its training framework also incorporates Progressive Loss, Small Target-Aware Label Assignment (STAL), and the MuSGD optimizer [25,26].

The zero-shot benchmark comprises YOLO-World, Grounding DINO, OWL-ViT, and OWLv2. These models represent distinct approaches to open-vocabulary detection. Grounding DINO integrates language representations with a transformer-based detector through cross-modal feature fusion and language-guided query selection. OWL-ViT adapts vision-language representations for text-conditioned object localization, while OWLv2 extends this framework through large-scale self-training. YOLO-World integrates visual-semantic representations into a YOLO-based framework for efficient open-vocabulary detection.

All zero-shot models were evaluated without domain-specific fine-tuning. Text prompts representing underground personnel were specified according to the prompting interface of each model. Model-specific confidence thresholds were selected exclusively from the validation subset and then fixed for the held-out test evaluation. For models using multiple personnel-related prompts, the resulting detections were mapped to a common personnel class. Class-agnostic non-maximum suppression (NMS) at an intersection-over-union (IoU) threshold of 0.50 was subsequently applied to suppress duplicate predictions arising from the merged detections. This procedure standardized the zero-shot outputs into a common single-class detection format for comparison with the ground-truth miner annotations.

### 3.3. Experimental Setup

All fine-tuned YOLO detectors were trained under a controlled experimental configuration to support consistent comparison across model architectures. Training was conducted on a high-performance computing cluster using an NVIDIA A100 80 GB PCIe GPU configured as a 3g.40gb Multi-Instance GPU (MIG) partition. Each model was trained for a maximum of 200 epochs using 640 × 640-pixel input images and a batch size of 32. Early stopping was applied after 50 consecutive epochs without improvement in validation performance. An initial learning rate of 0.01 was used for all models, with Adam for YOLOv8s, YOLOv9s, and YOLO11s, and MuSGD for YOLO26s. Four data-loading workers and a random seed of 42 were used across all training runs. Table 5 summarizes the principal training hyperparameters and computational settings used for the fine-tuned YOLO detectors.

**Figure 4:**
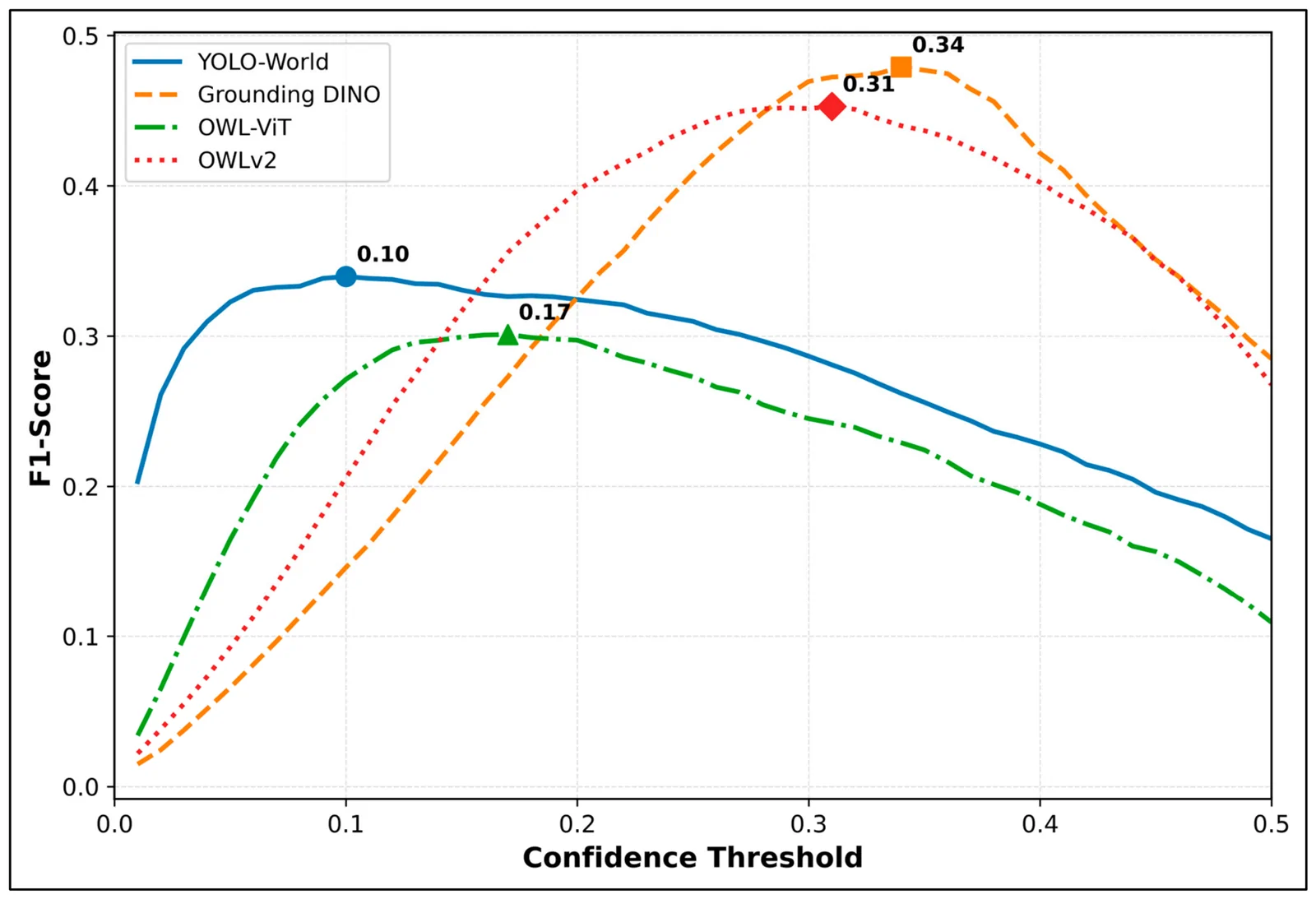
F1-score sensitivity of the zero-shot models across confidence thresholds on the validation subset.

The zero-shot vision-language models were evaluated using pretrained weights without task-specific fine-tuning on the underground coal mine dataset. A common set of personnel-related concepts comprising “person,” “worker,” “miner,” and “coal miner” was used to define the detection target. The textual formulation was adapted to the prompting interface of each model, as summarized in Table 6. Model-specific confidence thresholds were selected exclusively on the validation subset by maximizing the F1-score over a predefined threshold search space. Figure 4 shows the variation in F1-score across the evaluated confidence thresholds and identifies the validation-selected operating point for each zero-shot model. The selected thresholds were 0.10 for YOLO-World, 0.34 for Grounding DINO, 0.17 for OWL-ViT, and 0.31 for OWLv2. These thresholds were subsequently fixed for evaluation on the held-out test subset. This procedure prevented the use of test data during confidence-threshold selection.

Computational performance was assessed using the same NVIDIA A100 80 GB PCIe GPU configured as a 3g.40gb MIG partition for all eight models. For the fine-tuned YOLO detectors, inference time was obtained from the Ultralytics evaluation framework using a batch size of 16 and excluded preprocessing and postprocessing. The zero-shot models were evaluated sequentially at one image per inference call using their respective inference pipelines. Model throughput was calculated from the reported inference time and expressed in frames per second (FPS). Because the timing procedures differed between the fine-tuned and zero-shot pipelines, the reported latency and throughput values are treated as hardware-controlled computational reference measurements rather than strictly standardized end-to-end latency comparisons.

### 3.4. Evaluation Metrics

Detection performance was evaluated using standard object detection metrics, applied consistently across both detection paradigms. Predicted detections were matched to ground-truth annotations using an Intersection-over-Union (IoU) threshold of 0.50. A prediction was classified as a true positive (*TP*) if its IoU with an unmatched ground-truth annotation was at least 0.50. Unmatched predictions were classified as false positives (*FP*s), and unmatched ground-truth annotations were classified as false negatives (*FN*s).

Precision is the proportion of predicted detections that are correct. Recall is the proportion of ground-truth miners that are successfully detected. The F_1_-score is the harmonic mean of precision and recall, providing a balanced assessment of both metrics. Equations (1)–(3) define these metrics.

(1) $$\mathrm{Precision}=\frac{TP}{TP+FP}$$

(2) $$\mathrm{Recall}=\frac{TP}{TP+FN}$$

(3) $$F_{1}=\frac{2\times \mathrm{Precision}\times \mathrm{Recall}}{\mathrm{Precision}+\mathrm{Recall}}$$

where *TP*, *FP*, and *FN* denote the counts of true positive, false positive, and false negative detections, respectively.

Detection performance was further evaluated using Average Precision at an IoU threshold of 0.50 (*AP*_50_) and mean Average Precision across IoU thresholds from 0.50 to 0.95 (*AP*_50–95_). Precision–recall curves and average precision were derived from confidence-ranked detections rather than from predictions at a single operating confidence threshold. Predictions were ranked in descending order of confidence and cumulatively matched to ground-truth annotations to construct the precision–recall curve. *AP*_50_ was defined as the area under the precision–recall curve at an IoU threshold of 0.50, as expressed in Equation (4):

(4) $$AP_{50}=\int_{0}^{1}P_{0.50\left(R\right)dR}$$

where *P*_0.50_(*R*) denotes precision as a function of recall at an IoU threshold of 0.50. *AP*_50–95_ was calculated by averaging AP across IoU thresholds from 0.50 to 0.95 in increments of 0.05, as defined in Equation (5):

(5) $$AP_{50-95}=\frac{1}{10}\sum_{IoU\in \{0.50,0.55,\ldots ,0.95\}}AP\left(IoU\right)$$

*AP*_50_ was computed using the standard Ultralytics evaluation pipeline for the fine-tuned YOLO detectors and an equivalent confidence-ranked evaluation procedure for the zero-shot vision-language models. Precision, recall, and *F*_1_-score for the zero-shot models were reported at model-specific confidence thresholds selected exclusively on the validation subset and fixed prior to evaluation on the held-out test set. The corresponding metrics for the fine-tuned YOLO detectors were obtained using the standard Ultralytics evaluation procedure. Recall is of particular importance in underground personnel detection because false negatives correspond to miners that remain undetected by the perception system. Precision and *F*_1_-score complement recall by characterizing false-alarm behavior and the balance between detection sensitivity and precision, while *AP*_50_ and *AP*_50–95_ provide broader measures of detection and localization performance across confidence and IoU criteria. Inference time and frames per second (FPS) were additionally reported as computational performance indicators under the common benchmark hardware.

For the target-scale analysis, apparent personnel target size was defined from the distribution of ground-truth bounding-box areas in the held-out test set. The 3408 ground-truth miner instances were ranked by bounding-box area and divided into three equal-frequency tertiles corresponding to small, medium, and large apparent target scales, with 1136 instances in each category. This data-driven categorization produced balanced scale-specific evaluation subsets without imposing arbitrary fixed pixel-area thresholds. The resulting categories represent the relative image-space size of personnel targets within the test set and do not correspond to the miners’ physical dimensions or measured distance from the camera. Scale-specific recall for each detector was computed by matching predicted detections to ground-truth miner instances at an IoU threshold of 0.50 and determining the proportion of ground-truth instances successfully detected within each target-scale category.

## 4. Results

Table 7 summarizes the performance of the fine-tuned YOLO detectors and zero-shot vision-language models (VLMs) on the held-out underground mine test set. Performance is reported using precision, recall, F1-score, AP50, AP50–95, inference time, and frames per second (FPS). These metrics characterize detection performance, localization quality, and computational performance. The results are organized according to the two detection paradigms evaluated in this study. Section 4.1 presents the performance of the fine-tuned YOLO detectors, Section 4.2 reports the zero-shot VLM results, and Section 4.3 provides a direct comparison between the two paradigms under the common evaluation protocol.

### 4.1. Fine-Tuned YOLO Model Results

**Figure 5:**
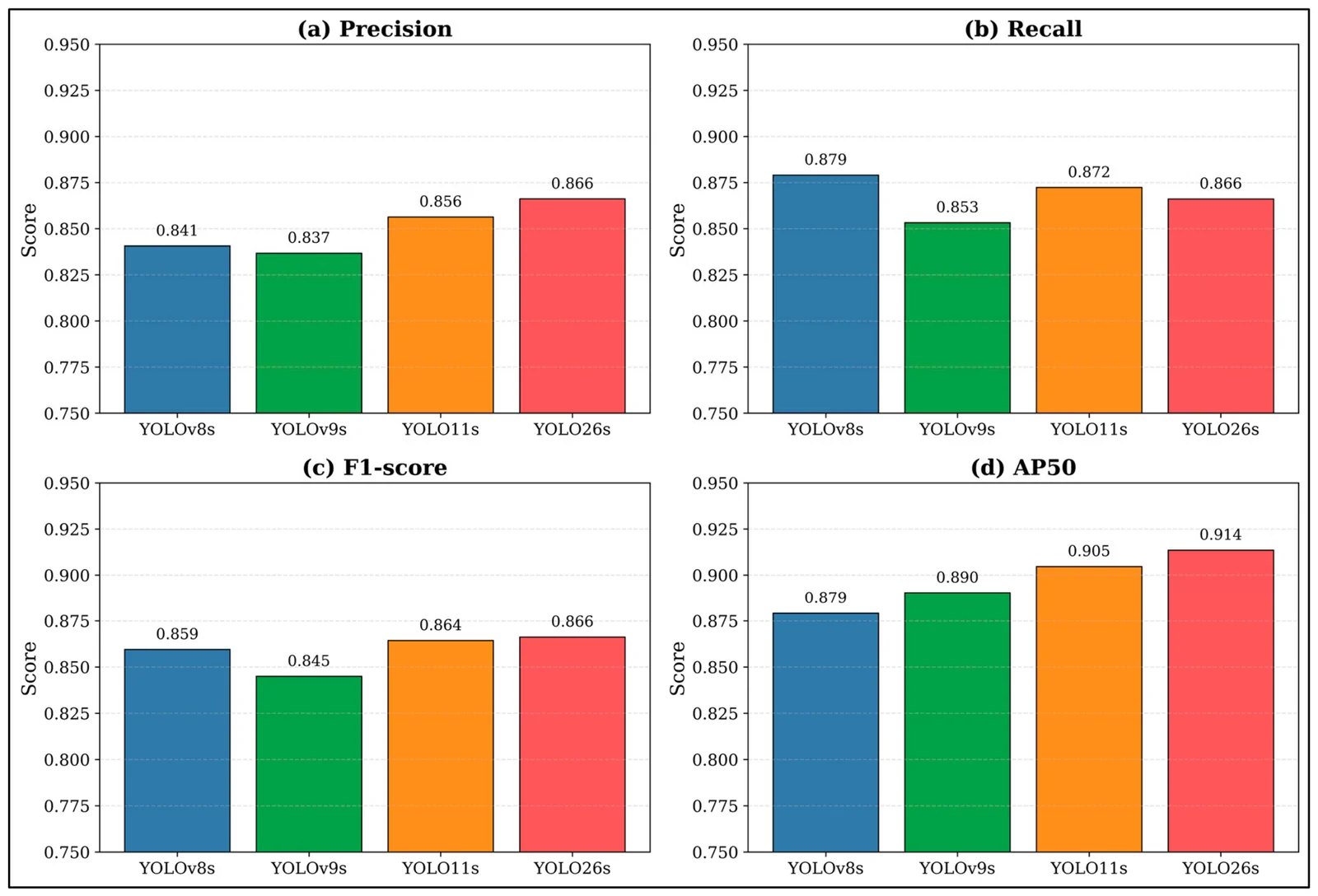
Performance comparison of fine-tuned YOLO detectors for personnel detection.

**Figure 6:**
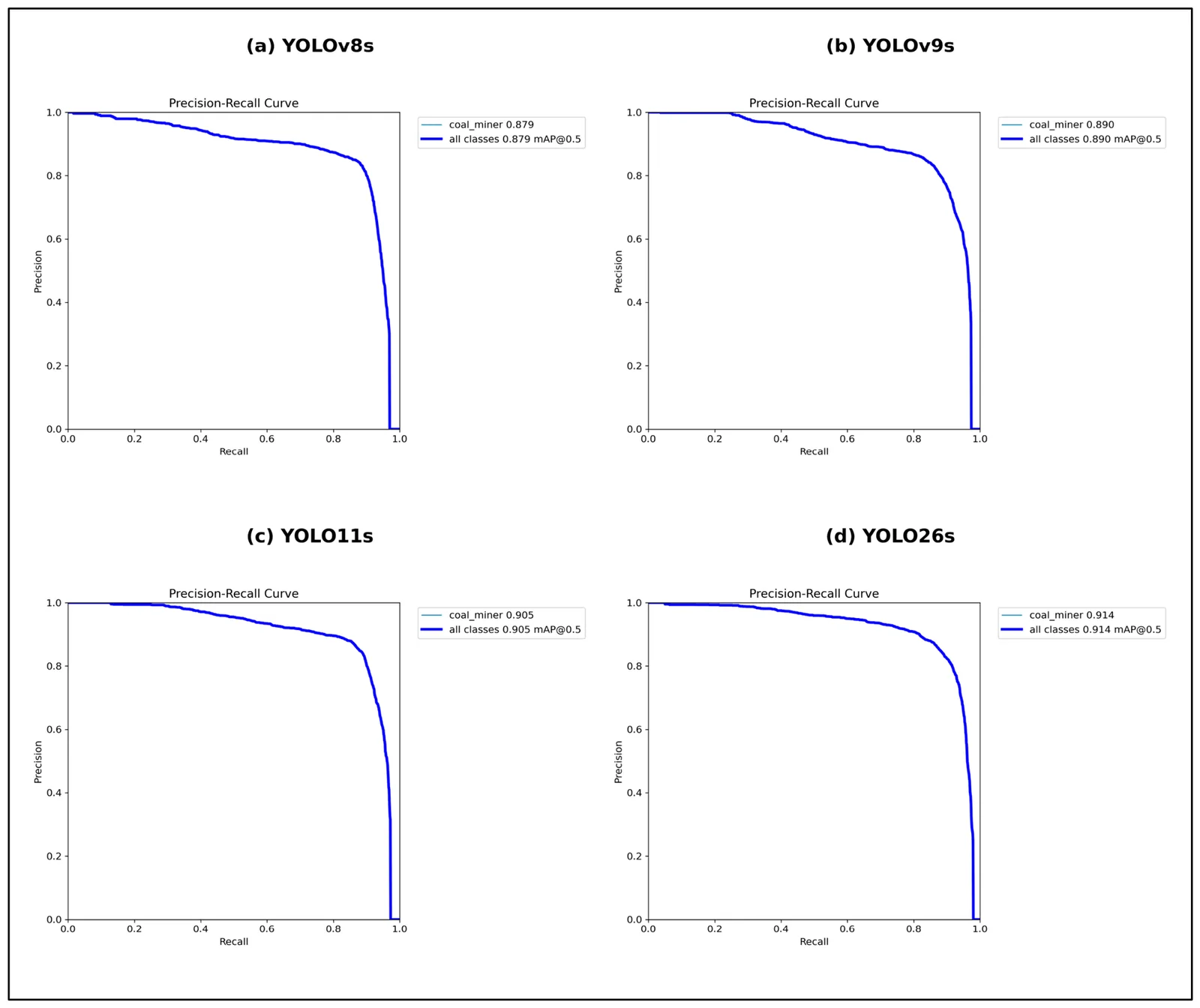
Precision–recall curves for fine-tuned YOLO detectors for personnel detection.

All four fine-tuned YOLO detectors achieved strong personnel-detection performance on the held-out underground mine test set. Precision ranged from 0.8368 to 0.8662, recall from 0.8533 to 0.8791, and F1-score from 0.8449 to 0.8662. AP50 ranged from 0.8791 to 0.9135, while AP50–95 ranged from 0.4203 to 0.4563. Inference times were consistently low, ranging from 1.30 to 1.80 ms per image. YOLO26s achieved the strongest overall supervised performance, recording the highest precision (0.8662), F1-score (0.8662), AP50 (0.9135), and AP50–95 (0.4563). YOLOv8s achieved the highest recall (0.8791), while YOLO11s also performed strongly with an F1-score of 0.8643 and AP50 of 0.9046. YOLOv9s recorded an F1-score of 0.8449 and AP50 of 0.8904. Figure 5 compares precision, recall, F1-score, and AP50 across the four fine-tuned detectors. The models remained relatively close across the principal detection metrics. YOLO26s achieved the highest precision, F1-score, and AP50, while YOLOv8s achieved the highest recall. Figure 6 presents the corresponding precision–recall curves, with the AP50 ranking of YOLO26s, YOLO11s, YOLOv9s, and YOLOv8s matching the quantitative results reported in Table 7.

### 4.2. Zero-Shot Vision-Language Model Results

**Figure 7:**
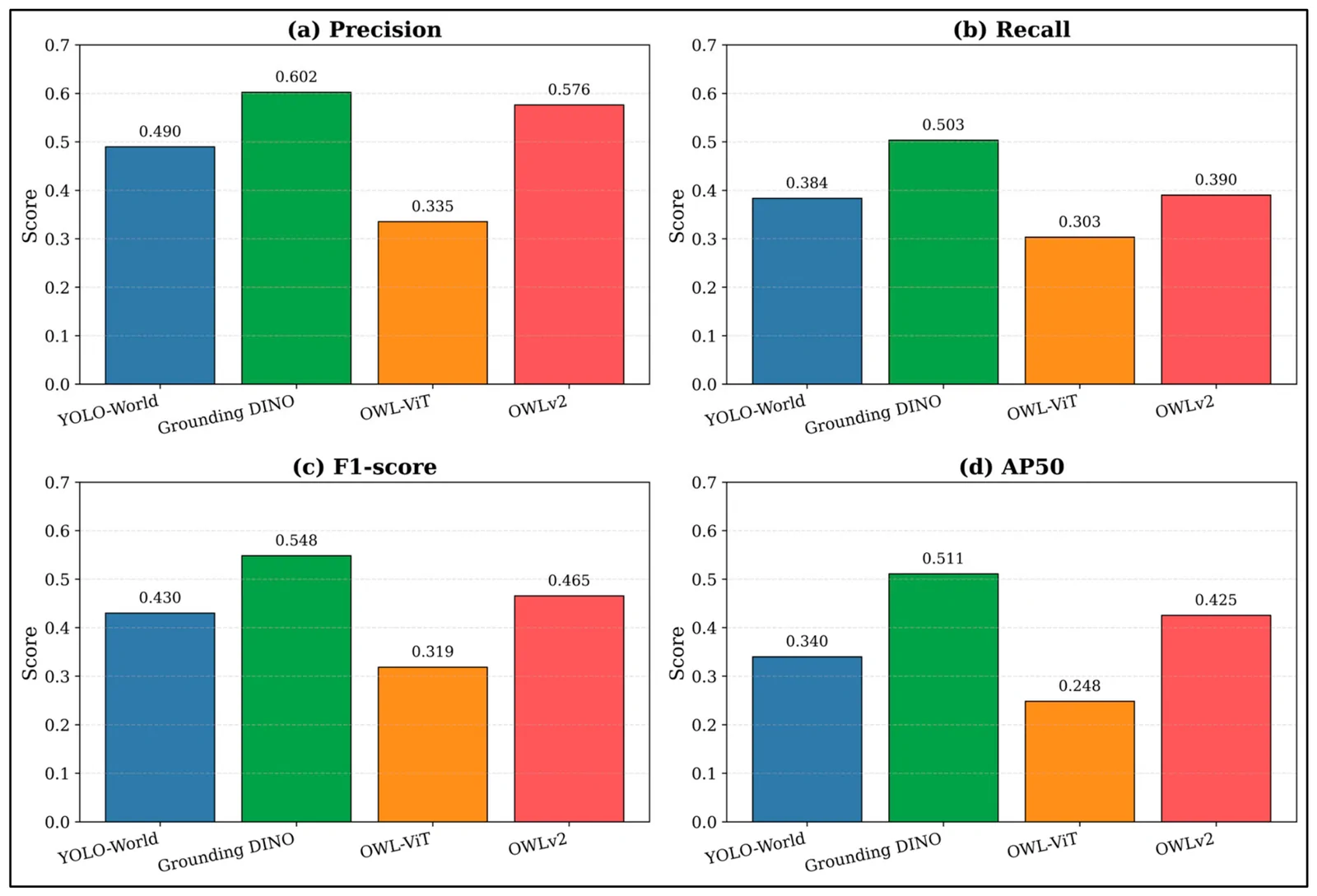
Performance comparison of the zero-shot vision-language models.

**Figure 8:**
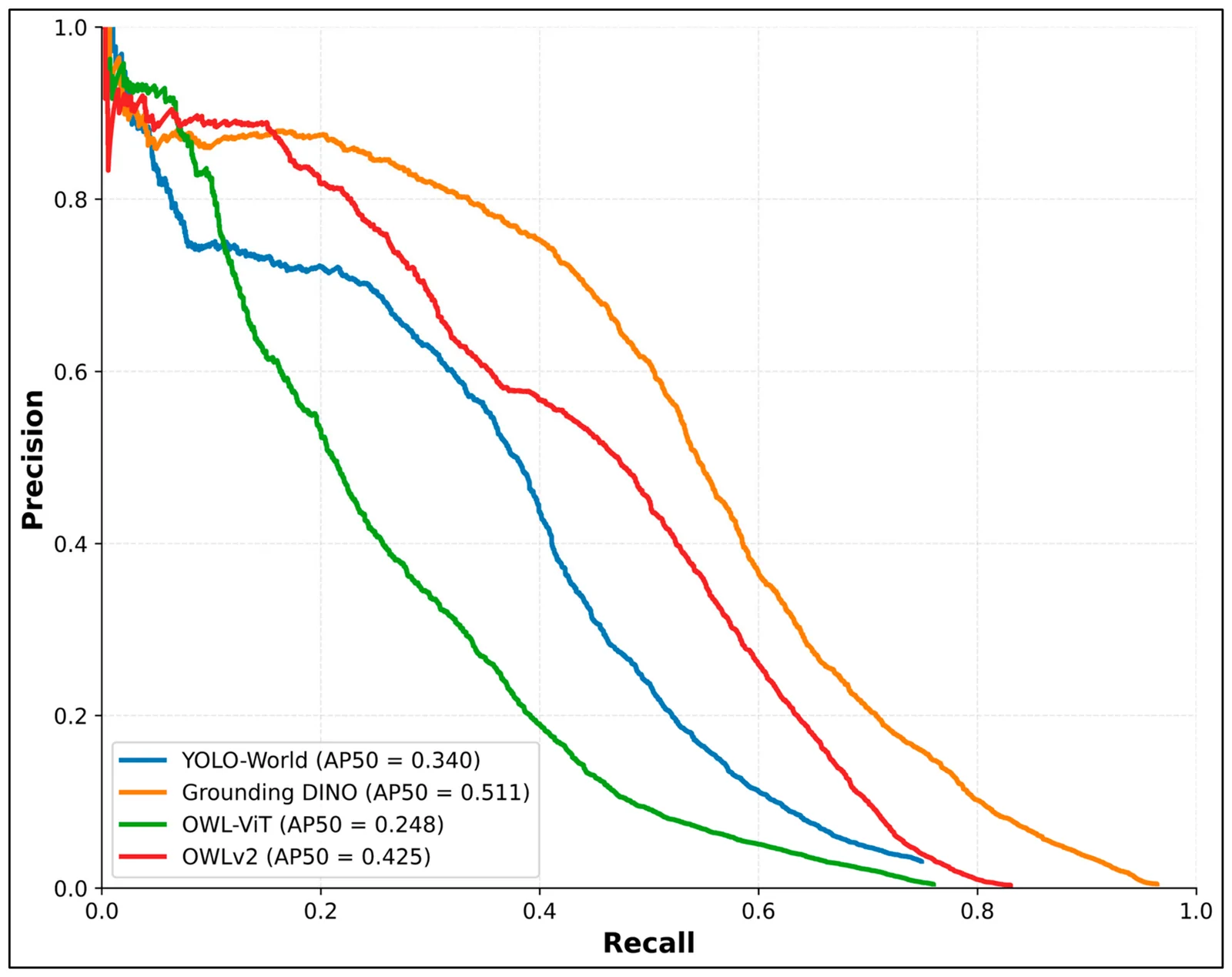
Precision–recall curves for zero-shot vision-language models for personnel detection.

As shown in Table 7, the zero-shot vision-language models exhibited substantial variation in detection performance. Precision ranged from 0.3355 to 0.6024, recall from 0.3034 to 0.5032, F1-score from 0.3186 to 0.5484, AP50 from 0.2484 to 0.5108, and AP50–95 from 0.0867 to 0.1830. Grounding DINO achieved the strongest overall zero-shot performance, recording the highest precision (0.6024), recall (0.5032), F1-score (0.5484), AP50 (0.5108), and AP50–95 (0.1830). OWLv2 ranked second, with a precision of 0.5763, F1-score of 0.4654, AP50 of 0.4254, and AP50–95 of 0.1490. YOLO-World achieved an F1-score of 0.4301 and AP50 of 0.3400, while OWL-ViT recorded the lowest F1-score (0.3186), AP50 (0.2484), and AP50–95 (0.0867). Measured inference throughput also varied considerably across the evaluated zero-shot models. YOLO-World achieved the highest throughput at 81.05 FPS with an average inference time of 12.34 ms per image, followed by OWL-ViT at 35.81 FPS. OWLv2 achieved 6.01 FPS, while Grounding DINO recorded the lowest throughput at 2.35 FPS with an average inference time of 425.32 ms per image. Figure 7 summarizes precision, recall, F1-score, and AP50 across the four models. Grounding DINO achieved the highest values across all four detection metrics, while YOLO-World provided the highest computational throughput. Figure 8 presents the precision–recall curves for the four zero-shot vision-language models. Grounding DINO achieved the highest AP50 of 0.5108, followed by OWLv2 at 0.4254, YOLO-World at 0.3400, and OWL-ViT at 0.2484. The precision–recall curves show a clear performance separation among the zero-shot models, with Grounding DINO maintaining the strongest overall precision–recall performance.

### 4.3. Comparative Benchmark Analysis

The cross-paradigm comparison showed a substantial performance advantage for the fine-tuned YOLO detectors. Their F1-scores ranged from 0.8449 to 0.8662, whereas the zero-shot vision-language models achieved 0.3186–0.5484. This difference was also evident in average precision. The fine-tuned detectors achieved AP50 values of 0.8791–0.9135, compared with 0.2484–0.5108 for the zero-shot models. Under the more stringent AP50–95 metric, the fine-tuned detectors achieved 0.4203–0.4563, while the zero-shot models achieved 0.0867–0.1830. The performance advantage of the fine-tuned detectors was therefore consistent across F1-score, AP50, and AP50–95.

**Figure 9:**
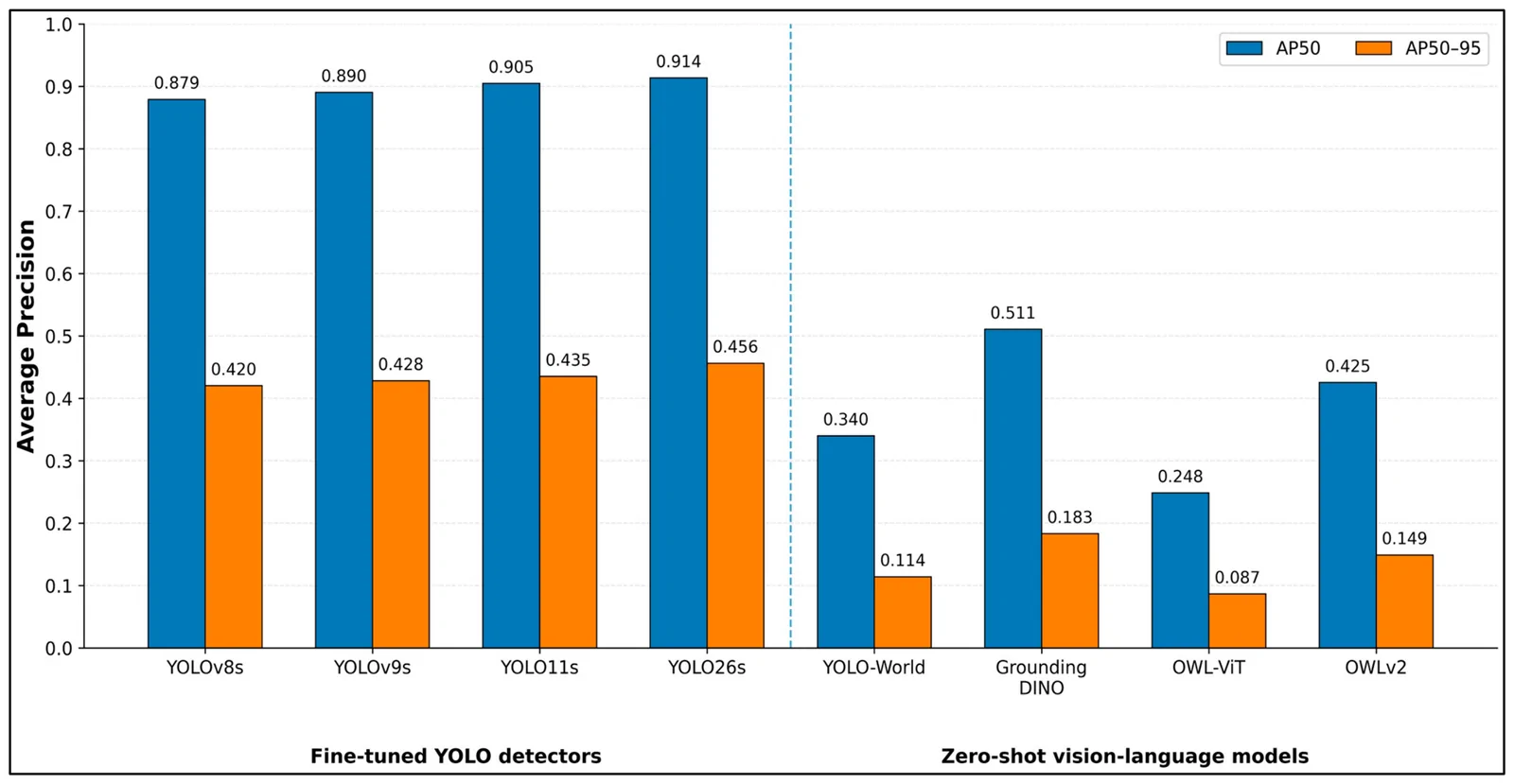
Cross-paradigm comparison of AP50 and AP50–95 for the evaluated fine-tuned and zero-shot models. The dotted line separates the two detection paradigms.

Figure 9 provides a direct comparison of AP50 and AP50–95 across all eight models. YOLO26s achieved the highest values, with an AP50 of 0.9135 and an AP50–95 of 0.4563. Grounding DINO achieved the strongest zero-shot performance, with corresponding values of 0.5108 and 0.1830. However, its performance remained below that of every fine-tuned detector. The lowest AP50 among the fine-tuned models was 0.8791, compared with 0.5108 for Grounding DINO. The same pattern was observed for AP50–95, with respective values of 0.4203 and 0.1830. These results demonstrate a clear separation between the two paradigms under both IoU evaluation criteria.

**Figure 10:**
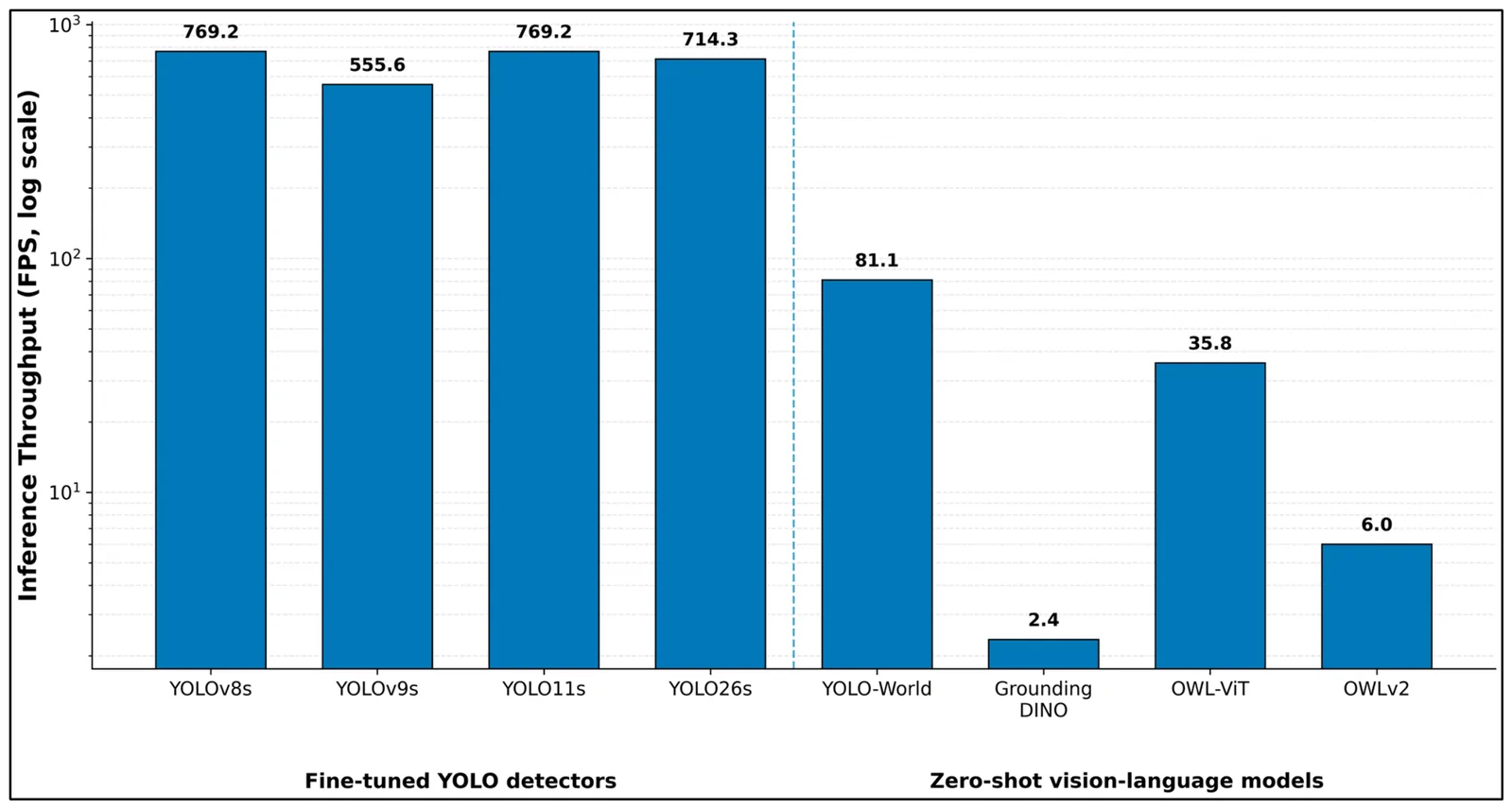
Inference throughput of fine-tuned YOLO detectors and zero-shot vision-language models on the common benchmark hardware. The dotted line separates the two detection paradigms.

The measured inference throughput also differed substantially across the evaluated models. As shown in Figure 10, the fine-tuned YOLO detectors recorded throughput between 555.56 and 769.23 FPS, while the zero-shot models recorded 2.35–81.05 FPS. YOLO-World achieved the highest throughput among the zero-shot models at 81.05 FPS, while Grounding DINO recorded the lowest at 2.35 FPS. These measurements were obtained using the same GPU configuration, although the fine-tuned and zero-shot models were evaluated through different model-specific inference pipelines and batch settings. The results therefore provide hardware-controlled computational reference measurements and should not be interpreted as strictly standardized end-to-end latency comparisons between the two paradigms.

**Figure 11:**
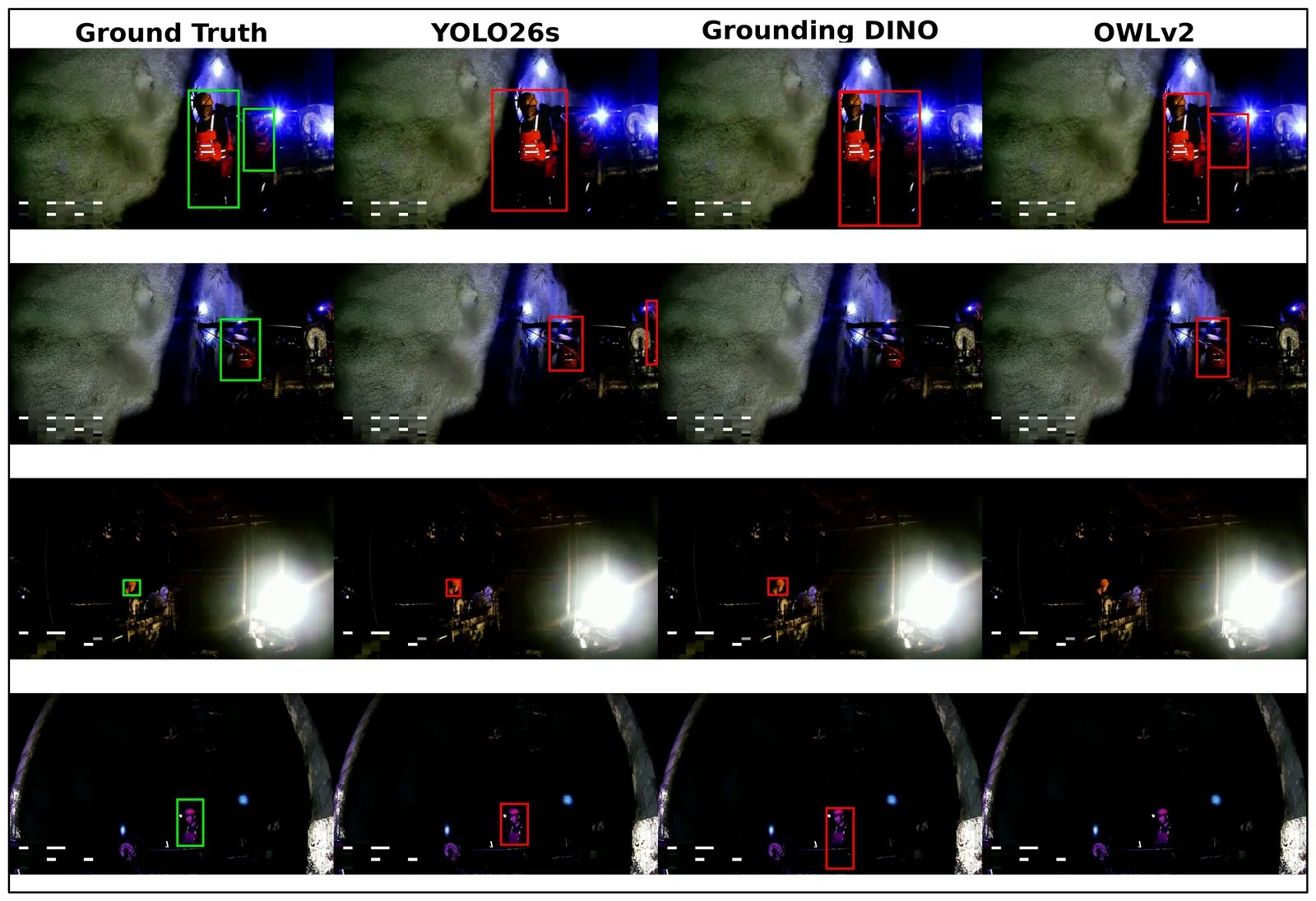
Qualitative comparison of underground personnel detection across representative test scenes. Green boxes indicate ground-truth annotations, while red boxes indicate model predictions.

Figure 11 presents representative qualitative detection results from the held-out test set. The figure compares ground-truth annotations with predictions from YOLO26s, Grounding DINO, and OWLv2. In the selected examples, YOLO26s produced detections with close correspondence to the annotated miner locations. Grounding DINO successfully detected personnel in several scenes but also exhibited false-positive detections and localization errors. OWLv2 showed similar variability, including successful detections, missed personnel instances, and localization errors. The qualitative results further support the performance separation observed between the fine-tuned and zero-shot detection paradigms in the quantitative benchmark.

### 4.4. Condition-Specific and Target-Scale Performance

To further examine the cross-paradigm differences identified in Section 4.3, recall was evaluated across brightness, fuzziness, and occlusion conditions in the held-out test set, as summarized in Table 8. Recall was emphasized because false negatives correspond to missed personnel instances. Target-scale performance was also evaluated across small, medium, and large apparent personnel targets.

The fine-tuned detectors maintained substantially higher recall across the evaluated visual conditions. YOLO26s achieved the highest supervised recall under high brightness (0.802), mid brightness (0.899), fuzzy (0.929), non-fuzzy (0.878), occluded (0.920), and non-occluded (0.894) conditions. YOLOv8s achieved the highest supervised recall under low brightness at 0.945. Among the zero-shot models, Grounding DINO achieved the highest recall in six of the seven condition categories. OWLv2 achieved the highest zero-shot recall under high brightness at 0.626. A pronounced cross-paradigm difference was observed for occluded personnel. YOLO26s achieved a recall of 0.920, compared with 0.386 for Grounding DINO. This represents a 53.4 percentage-point difference between the leading models from the two paradigms.

The condition-specific analysis reflects the visual characteristics present within the held-out test set. Brightness, fuzziness, and occlusion can coexist within the same scene; therefore, the condition subsets are not mutually exclusive. The reported recall values quantify model performance under each visual condition without attributing the observed performance differences to any single environmental factor. Target-scale performance was further examined to determine how recall varied with the apparent image-space size of personnel targets. Table 9 summarizes recall for small, medium, and large apparent personnel targets across the two detection paradigms.

Target-scale analysis revealed a substantial performance separation between the two detection paradigms. Small apparent personnel targets produced the lowest recall for all eight models. Fine-tuned recall for small targets ranged from 0.816 to 0.864, compared with 0.172–0.386 for the zero-shot models. YOLO26s achieved the highest supervised recall across small (0.864), medium (0.939), and large (0.913) targets. Grounding DINO achieved the highest zero-shot recall across the same target scales, with corresponding values of 0.386, 0.614, and 0.511. The largest cross-paradigm difference was observed for small apparent targets. YOLO26s exceeded Grounding DINO by 47.8 percentage points in this category. The fine-tuned detectors maintained higher recall than all zero-shot models across each of the three target-scale categories.

## 5. Discussion

The benchmark results show a clear performance advantage for fine-tuned YOLO detectors over zero-shot vision-language models in underground mine personnel detection. All four supervised detectors outperformed the zero-shot models across the principal detection metrics. The supervised models achieved F1-scores of 0.8449–0.8662, compared with 0.3186–0.5484 for the zero-shot models. AP50 followed the same pattern. The supervised models achieved AP50 values of 0.8791–0.9135, whereas the zero-shot models achieved 0.2484–0.5108. The consistent separation across F1-score, AP50, and AP50–95 indicates that the performance difference extends beyond a single operating threshold or evaluation criterion. Within the evaluated underground domain, the fine-tuned detectors benefited from task-specific adaptation to personnel appearance and scene characteristics. The zero-shot models offer greater flexibility because they can recognize semantic categories without task-specific training. However, this flexibility did not translate into comparable detection performance on the underground dataset.

The condition-specific and target-scale analyses provide further insight into this performance gap. Fine-tuned detectors maintained higher recall across the evaluated brightness, fuzziness, and occlusion conditions. The largest observed recall difference occurred for occluded personnel. YOLO26s achieved a recall of 0.920 for occluded personnel, compared with 0.386 for Grounding DINO. This represents a 53.4 percentage-point difference. Target-scale analysis revealed a similarly pronounced separation between the two detection paradigms. Small apparent personnel targets were particularly challenging for the zero-shot models. Recall for small targets ranged from 0.816 to 0.864 among the supervised detectors and from 0.172 to 0.386 among the zero-shot models. YOLO26s achieved a small-target recall of 0.864, whereas Grounding DINO achieved 0.386. The resulting difference was 47.8 percentage points. These results show that the performance advantage of the fine-tuned detectors was maintained under more demanding detection scenarios, particularly when personnel were occluded or occupied a small region of the image. The condition-specific results also demonstrate that this advantage was consistent across the range of visual conditions represented in the underground dataset.

Among the supervised detectors, YOLO26s achieved the strongest overall performance. It recorded the highest precision, F1-score, AP50, and AP50–95, while YOLOv8s achieved the highest recall. The relatively close performance of the supervised models indicates that all four architectures performed strongly after domain-specific training. Among the zero-shot models, Grounding DINO achieved the strongest overall detection performance, whereas YOLO-World provided substantially higher inference throughput but lower detection accuracy. This result indicates an accuracy–throughput trade-off among the zero-shot detectors. Grounding DINO achieved the highest zero-shot F1-score and AP50 but operated at only 2.35 FPS. In contrast, YOLO-World reached 81.05 FPS but produced lower F1-score and AP50 values of 0.4301 and 0.3400, respectively. This contrast demonstrates that the strongest zero-shot detection performance and the highest zero-shot computational throughput were achieved by different architectures. However, even the strongest zero-shot detection performance remained substantially below that of all four fine-tuned detectors. The results therefore indicate that domain-specific fine-tuning currently provides stronger detection performance within the evaluated underground personnel-detection task than direct zero-shot inference.

### 5.1. Implications for Underground Autonomous Haulage Perception

The results have important implications for designing personnel-perception systems for underground autonomous haulage. Fine-tuned YOLO detectors outperformed the zero-shot models across the evaluated metrics and visual conditions. This distinction is important because personnel detection is an upstream component of the autonomous perception and decision pipeline. Missed detections can prevent personnel information from reaching downstream functions such as proximity assessment, collision-risk evaluation, warning generation, and vehicle-response logic. The substantially higher recall of the fine-tuned detectors therefore corresponds to a lower proportion of missed personnel instances within the evaluated dataset. This distinction was especially pronounced for occluded and small apparent personnel targets. For safety-critical personnel detection, this is particularly important because high overall accuracy alone does not ensure that personnel are consistently detected across challenging operating conditions.

The results also highlight the need to consider detection performance and computational efficiency together. An autonomous perception system must process sensor data within the timing requirements of downstream decision and control functions. However, high inference throughput has limited operational value if it is accompanied by frequent missed detections. Conversely, high detection performance must be achievable within the available onboard computational resources and latency constraints. The benchmark therefore indicates that personnel-detection models for underground autonomous haulage should be evaluated as a combined accuracy–robustness–efficiency problem. In addition to conventional precision and average precision, particular attention should be given to recall, false-negative behavior, performance under challenging conditions, target-scale sensitivity, and inference latency.

### 5.2. Limitations and Future Work

The present benchmark provides a controlled comparison of fine-tuned and zero-shot detectors using an underground coal mine image dataset. However, the evaluation focuses on personnel detection within a single underground mining domain. Variations in mine geometry, illumination, equipment, infrastructure, and operating conditions may affect model performance across different sites. The single-class design also focuses the benchmark on the safety-critical task of personnel detection rather than broader multi-class scene perception. Evaluation across additional underground mines and operationally relevant object classes would provide a broader assessment of model generalization while extending the benchmark to more diverse underground environments.

The zero-shot evaluation also depends on the textual prompts used to define the personnel class. Common personnel-related concepts were applied across the models, with their formulation adapted to each model’s prompting interface. Confidence thresholds were selected exclusively on the validation subset and fixed before test evaluation. However, alternative prompt formulations may influence detection performance. Future work will systematically evaluate prompt sensitivity for underground personnel detection.

The present study focuses on RGB-based perception, while computational performance was evaluated under a common NVIDIA A100 MIG configuration. The common hardware configuration provided a consistent basis for computational assessment, with latency and throughput reported according to the respective inference pipelines and batch settings of the two detection paradigms. Future work will extend the benchmark through cross-mine and multi-class evaluation, synchronized RGB–LiDAR perception, and evaluation on representative onboard computing platforms. These extensions will support broader assessment of perception performance for underground autonomous haulage systems.

## 6. Conclusions

This study established a cross-paradigm benchmark for underground mine personnel detection by comparing four fine-tuned YOLO detectors with four zero-shot vision-language models under a common experimental framework. The results showed a consistent performance advantage for the fine-tuned detectors in detection accuracy and localization quality. The fine-tuned models also recorded substantially higher inference throughput under the common benchmark hardware. YOLO26s achieved the strongest overall fine-tuned performance, while Grounding DINO was the strongest zero-shot model. The findings demonstrate that domain-specific fine-tuning currently provides stronger performance than direct zero-shot inference for the evaluated underground personnel-detection task.

The condition-specific and target-scale analyses further revealed important differences between the two detection paradigms. Fine-tuned detectors maintained higher recall across brightness, fuzziness, and occlusion conditions and across apparent target scales. The performance separation was particularly pronounced for occluded personnel and small apparent targets. These findings highlight the importance of evaluating underground personnel detectors across challenging visual conditions and target scales, particularly where missed detections can affect the reliability of personnel perception.

The benchmark provides quantitative baselines for comparing fine-tuned and zero-shot detection approaches for underground personnel perception. It also demonstrates the value of combining conventional detection metrics with condition-specific, target-scale, and computational evaluation. Future work will extend the benchmark across additional underground mines and operationally relevant object classes, together with systematic prompt analysis, synchronized RGB–LiDAR perception, and evaluation on representative onboard computing platforms. These extensions will support more comprehensive evaluation of robust perception systems for underground autonomous haulage.

## Figures and Tables

**Table 1:** Dataset split statistics for the underground coal mine benchmark dataset.

Split	Images	Proportions (%)	Use in Benchmark
Train	25,117	80.00	Fine-tuned YOLO model training
Validation	3140	10.00	Model tuning and zero-shot threshold selection
Test	3139	10.00	Final comparative evaluation
**Total**	**31,396**	**100.00**	

Note: Bold text and values indicate the overall dataset total.

**Table 2:** Architectural and computational characteristics of the benchmark models.

Model	Paradigm	Key Architectural Feature	Parameters (M)
YOLOv8s	Fine-tuned	C2f feature extraction; anchor-free detection	11.14
YOLOv9s	Fine-tuned	GELAN; Programmable Gradient Information	7.29
YOLO11s	Fine-tuned	C3k2 modules; C2PSA attention	9.43
YOLO26s	Fine-tuned	End-to-end NMS-free detection; DFL-free box regression	9.95
YOLO-World	Zero-shot	Vision-language alignment within a YOLO detection framework	13.38
Grounding DINO	Zero-shot	Transformer-based detection with language grounding	172.25
OWL-ViT	Zero-shot	Vision Transformer-based open-vocabulary detection	153.23
OWLv2	Zero-shot	Scalable open-vocabulary detection with self-training	154.97

**Table 3:** Architectural comparison of the fine-tuned YOLO detectors.

Model	Principal Feature-Extraction Architecture	Detection Head	Inference Strategy	Initialization	Key Characteristic
YOLOv8s	C2f-based backbone	Anchor-free	NMS-based	Pretrained weights	Efficient anchor-free detection
YOLOv9s	GELAN	Anchor-free	NMS-based	Pretrained weights	PGI-assisted information preservation
YOLO11s	C3k2 and C2PSA modules	Anchor-free	NMS-based	Pretrained weights	C2PSA-enhanced feature representation
YOLO26s	C3k2 and C2PSA-based architecture	One-to-one end-to-end head	NMS-free	Pretrained weights	Simplified end-to-end inference

**Table 4:** Architectural characteristics of the zero-shot vision-language models.

Model	Vision Encoder	Language Representation	Detection Strategy	Key Characteristic
YOLO-World	YOLO-based visual encoder	CLIP-derived text representations	Open-vocabulary detection	Real-time open-vocabulary detection
Grounding DINO	Swin Transformer	BERT-based language encoder	Text-guided grounding	Language-guided object localization
OWL-ViT	Vision Transformer	CLIP-style text encoder	Open-vocabulary detection	Text-conditioned open-vocabulary detection
OWLv2	Vision Transformer	Text encoder aligned with the OWL framework	Open-vocabulary detection	Large-scale self-trained open-vocabulary detection

**Table 5:** Training hyperparameters for the fine-tuned YOLO detectors.

Hyperparameter	Configuration
Maximum epochs	200
Batch size	32
Input image size	640 × 640 pixels
Optimizer	Adam (YOLOv8s, YOLOv9s, YOLO11s); MuSGD (YOLO26s)
Initial learning rate	0.01
Early stopping patience	50 epochs
Data-loading workers	4
Random seed	42
GPU	NVIDIA A100 80 GB PCIe (MIG 3g.40gb partition)
Framework	PyTorch 2.12.1 (CUDA 13.0); Ultralytics 8.4.71

**Table 6:** Zero-shot prompting and inference configuration.

Model	Text Prompts	Confidence Threshold	IoU Threshold
YOLO-World	“person”, “worker”, “miner”, “coal miner”	0.10	0.50
Grounding DINO	“person. worker. miner. coal miner.”	0.34	0.50
OWL-ViT	“a photo of a person”, “a photo of a worker”, “a photo of a miner”, “a photo of a coal miner”	0.17	0.50
OWLv2	“a photo of a person”, “a photo of a worker”, “a photo of a miner”, “a photo of a coal miner”	0.31	0.50

**Table 7:** Performance comparison of fine-tuned YOLO detectors and zero-shot vision-language models.

Model	Paradigm	Precision	Recall	F1	AP50	AP50–95	Inf. (ms)	FPS
YOLOv8s	Supervised	0.8407	**0.8791**	0.8595	0.8791	0.4203	1.30	769.23
YOLOv9s	Supervised	0.8368	0.8533	0.8449	0.8904	0.4282	1.80	555.56
YOLO11s	Supervised	0.8564	0.8724	0.8643	0.9046	0.4351	1.30	**769.23**
YOLO26s	Supervised	**0.8662**	0.8662	**0.8662**	**0.9135**	**0.4563**	1.40	714.29
YOLO-World	Zero-shot	0.4897	0.3835	0.4301	0.3400	0.1141	**12.34**	81.05
Grounding DINO	Zero-shot	**0.6024**	**0.5032**	**0.5484**	**0.5108**	**0.1830**	425.32	2.35
OWL-ViT	Zero-shot	0.3355	0.3034	0.3186	0.2484	0.0867	27.93	35.81
OWLv2	Zero-shot	0.5763	0.3903	0.4654	0.4254	0.1490	166.42	6.01

Note: Bold values indicate the best-performing result for each metric within each detection paradigm (highest for Precision, Recall, F1, AP50, AP50–95, and FPS; lowest for inference time).

**Table 8:** Condition-specific recall across detection paradigms.

Model	Paradigm	High Brightness	Mid Brightness	Low Brightness	Fuzzy	Non-Fuzzy	Occluded	Non-Occluded
YOLOv8s	Supervised	0.746	0.859	**0.945**	0.899	0.869	0.899	0.875
YOLOv9s	Supervised	0.770	0.856	0.932	0.890	0.871	0.889	0.875
YOLO11s	Supervised	0.709	0.840	0.931	0.892	0.836	0.870	0.863
YOLO26s	Supervised	**0.802**	**0.899**	0.939	**0.929**	**0.878**	**0.920**	**0.894**
YOLO-World	Zero-shot	0.477	0.306	0.430	0.373	0.396	0.290	0.457
Grounding DINO	Zero-shot	0.579	**0.445**	**0.536**	**0.482**	**0.528**	**0.386**	**0.596**
OWL-ViT	Zero-shot	0.477	0.213	0.341	0.308	0.298	0.194	0.390
OWLv2	Zero-shot	**0.626**	0.420	0.302	0.321	0.471	0.295	0.466

Note: Bold values indicate the highest condition-specific recall within each detection paradigm.

**Table 9:** Recall performance across apparent personnel target scales.

Model	Paradigm	Small	Medium	Large
YOLOv8s	Supervised	0.836	0.915	0.905
YOLOv9s	Supervised	0.825	0.918	0.900
YOLO11s	Supervised	0.816	0.907	0.876
YOLO26s	Supervised	**0.864**	**0.939**	**0.913**
YOLO-World	Zero-shot	0.250	0.464	0.437
Grounding DINO	Zero-shot	**0.386**	**0.614**	**0.511**
OWL-ViT	Zero-shot	0.172	0.328	0.410
OWLv2	Zero-shot	0.313	0.391	0.467

Note: Bold values indicate the highest recall for each target scale within each detection paradigm.

## Data Availability

The dataset used in this study is publicly available as described in Reference [45].

## Abbreviations

The following abbreviations are used in this manuscript:

AHS	Autonomous Haulage Systems
CNN	Convolutional Neural Network
VLM	Vision-Language Model
YOLO	You Only Look Once
IoU	Intersection over Union
AP	Average Precision
AP50	Average Precision at an IoU threshold of 0.50
AP50–95	Mean Average Precision across IoU thresholds from 0.50 to 0.95
FPS	Frames Per Second
TP	True Positive
FP	False Positive
FN	False Negative
GPU	Graphics Processing Unit
NMS	Non-Maximum Suppression
MIG	Multi-Instance GPU
RGB	Red, Green, Blue
C2PSA	Cross-Stage Partial with Partial Self-Attention
CLIP	Contrastive Language–Image Pre-training
SGD	Stochastic Gradient Descent
MuSGD	Muon + SGD hybrid optimizer
